# Life History Theory and Social Psychology

**DOI:** 10.3389/fnevo.2012.00013

**Published:** 2012-09-03

**Authors:** Donald F. Sacco, Karol Osipowicz

**Affiliations:** ^1^The University of Southern MississippiHattiesburg, MS, USA; ^2^Department of Neurology, Thomas Jefferson UniversityPhiladelphia, PA, USA

While occupying a relatively brief space in the history of scientific inquiry, many social psychologists have nonetheless recognized the need to integrate the field’s myriad findings into a coherent, meta-theoretical framework. Chester et al. (2012), consistent with the spirit of this movement, have answered this call by utilizing a Life History Theory (LHT) framework to understand the relationship between attachment bonds formed during early critical developmental periods and later reactions to social rejection experiences in adulthood. While attachment theory and social exclusion research are well-developed phenomena in their own right, the utilization of LHT clarifies how these areas of inquiry are interrelated.

As the authors note, LHT has been successfully used across the biological sciences to explain how specific developmental periods require organisms to differentially allocate limited resources to navigate various aspects of survival and reproduction. At its core, LHT is a motivational framework, whereby motivational “states” are determined by the specific problems and opportunities associated with an organism’s current developmental stage and local ecology. In the current paper, Chester and colleagues provide a theoretical framework and supporting evidence indicating early life stages can critically influence, via calibration processes, responses in subsequent developmental periods.

**Figure 1:**
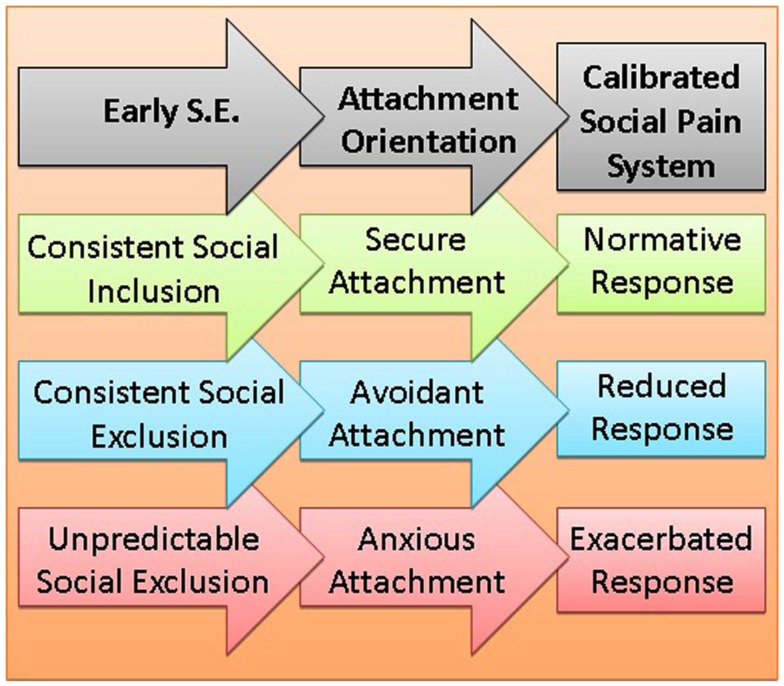
**Depicts the optimal calibration and response of the social pain system in relation to different life histories**. *Early S.E*. refers to the social environment in which the social pain system is calibrated, *Attachment Orientation* refers to the resultant attachment style produced by early attachment experiences, *Calibrated Social Pain System* refers to the sensitivity of the social pain system in response to social rejection in adulthood.

Consistent with the notion that evolution is incapable of forward-thinking, the authors argue that early social experiences provide the data that calibrates organisms’ systems for responding to their future social environments (see Figure 1). Specifically, children’s early attachment experiences calibrate their social pain system response, which partially determines the intensity of reactions to rejection experiences in adulthood. Although intense reactions to social rejection can be adaptive, specifically by helping an organism secure subsequent social belonging, the intensity of these responses is only beneficial provided social opportunity is actually available; otherwise, such chronic and intense responses to social rejection can negatively impact other vital systems (e.g., biological immune system). Whereas consistently unavailable attachment in childhood (avoidant attachment) communicates an environment in which social exclusion is likely to be normative, variable attachment opportunity in childhood (anxious attachment) indicates that social belonging opportunities will be unpredictable. Based on these early experiences, the authors provide evidence that avoidantly attached individuals show a blunted response to social rejection (reduced activation of dACC and insula), whereas anxiously attached individuals show a heightened response to social rejection. For avoidantly attached individuals, social resources have been consistently unavailable and calibration of the social pain system to respond intensely to social rejection would have numerous costs (e.g., suppression of immune system), with very few opportunities (social inclusion). Conversely, anxiously attached individuals, because social inclusion opportunities have been unpredictable, have a social pain system calibrated to be especially sensitive to cues signaling social rejection. Collectively, the current article effectively explains how early attachment bonds calibrate reactions to social rejection.

Interestingly, the *optimal calibration hypothesis* is not only a powerful explanatory framework for research on social attachment and social rejection; it may have clinical significance as well, with implications beyond social psychology. For example, an understanding of how the dACC and anterior insula are implicated in social pain system calibration may inform the understanding and treatment of individuals with damage to these areas, who are being treated primarily for symptoms unrelated to the social pain system. For example, patients with left middle cerebral artery (MCA) strokes often suffer injury to the anterior insula and the frontal opercula, producing pronounced expressive dysphasias. Some patients respond to therapy and recover expressive language function to almost basal levels, while other patients are completely intractable to therapy. While current treatments focus specifically on the recovery of language function, such injuries may also disrupt social pain responses, producing heightened levels of anxiety that can interfere with treatment adherence. If considered from a LHT perspective, this therapeutic approach should also address the patient’s attachment orientation and concomitant social pain system calibration to achieve optimal therapeutic outcomes. Understanding how specific brain injuries may disrupt social pain system responses could help catalyze the development of therapeutic interventions that address both primary (language deficits) and secondary symptoms (disruption of social pain system), which may improve overall patient outcomes. Thus, this theoretical framework has the potential to inform the study and treatment of neurologically impaired individuals.

Chester and colleagues also identify critical periods for the calibration of the social rejection system: (1) early on during development when parental rejection would be devastating to survival, and (2) during adolescence, when rejection of a possible mate would reduce reproductive fitness. Interestingly, these two critical periods are well aligned with critical periods in language development (e.g., Johnson and Newport, 1989; Hurford, 1991), suggesting that language and socio-affective development are inextricably linked – a postulation supported by the evolutionary evidence of con-committal language ability and societal size increases in hominids (Holloway, 1981). This allows for the extension of this hypothesis to not only include responses to social pain, but also linguistic function/ability as a calibratable system responsive to early life history. Interestingly, one of the major nodes of the social pain network (insula) is close in proximity and highly connected to a major node in the language network (Broca’s area), suggesting that the calibration of the social pain system and language system may be related. The development of these ideas could lead to interesting research that takes advantage of both neuropsychological and neuroimaging techniques.

Chester and colleagues have begun an exciting dialog that combines findings from a number of fields and unites them through one common theoretical perspective. Indeed, the current research program is one of a growing number in social psychology to use LHT to not only organize a variety of social psychological phenomena, but integrate them within the larger scientific community (e.g., Brown et al., 2009; Buss, 2009; Wang et al., 2009; Figueredo et al., 2011; Griskevicius et al., 2011; Sundie et al., 2011). Based on this expanding body of evidence, it seems as though a LHT framework will continue to be a powerful tool for organizing and generating rigorous research in the study of human behavior.
